# A National Study of Marital Status Differences in Early Uptake of COVID-19 Vaccine among Older Americans

**DOI:** 10.3390/geriatrics8040069

**Published:** 2023-06-28

**Authors:** Hui Liu, Gerald Roman Nowak, Juwen Wang, Zhehui Luo

**Affiliations:** 1 Department of Sociology, Michigan State University, 509 E. Circle Drive 316, East Lansing, MI 48824, USA; nowakge2@msu.edu (G.R.N.III); wangjuwe@msu.edu (J.W.); 2 Department of Epidemiology and Biostatistics, Michigan State University, East Lansing, MI 48824, USA; zluo@msu.edu

**Keywords:** COVID-19, vaccination, marital status, older adults

## Abstract

We provide one of the first nationally representative studies to examine COVID-19 vaccine uptake differences by marital status within the first year after the vaccine was recommended among older Americans. Data were drawn from the National Health and Aging Trends Study (2021). The study sample included 3180 participants aged 65 and older with 1846 women and 1334 men. Results from logistic regression models suggest that divorced/separated older adults were less likely to receive at least one dose of the COVID-19 vaccine in 2021 than their married counterparts, especially among women and individuals with higher education. Widowed and never married respondents were generally not significantly different from married respondents in COVID-19 vaccination status, with only one exception: less-educated never-married respondents were more likely to receive COVID-19 vaccination than their less-educated married counterparts. Our study highlights divorce/separation as a significant social factor associated with COVID-19 vaccine uptake among older adults in the U.S. These findings suggest that divorced/separated older adults are the most vulnerable population segment at risk of low COVID-19 vaccine uptake. Future efforts to improve vaccine equity and uptake should target this group specifically, with tailored interventions to increase their access and uptake of the vaccine.

## 1. Introduction

Leading governmental and public health agencies, such as the U.S. Centers for Disease Control (CDC) and World Health Organization (WHO), have consistently urged the general public to receive vaccine protection against the SARS-CoV-2 virus [1]. However, despite these efforts, approximately 17% of women and 22% of men in the U.S. had not received even a single dose of the primary COVID-19 vaccination series as of April 2023 [2]. Although vaccination coverage is relatively high among older Americans aged 65 and older, COVID-19 vaccination and booster uptake has plateaued and remains unequal across various social groups in the United States [3]. Given the elevated risk of COVID-related illness and death for those who remain unvaccinated, particularly among advanced age groups, it is crucial to identify vulnerable groups that are less likely to be vaccinated to improve public health outcomes during this global pandemic. In this study, we investigate marital status as a potential risk or protective factor for COVID-19 vaccination among older adults in the U.S.

Research has consistently shown that, compared to being unmarried, being married is associated with better health outcomes and better health behaviors, [4,5,6], while divorce and widowhood are associated with a range of worse outcomes, including worse self-rated health, worse cardiovascular health, higher risk of inflammation-related complications, and higher rates of cigarette use and alcohol consumption [7,8,9,10]. However, these marital advantages in health are not uniformly distributed across groups. For example, several studies have documented that marital status differences in both health and mortality are greater for men than for women, perhaps because men tend to receive greater health-promoting resources such as emotional support from a traditional marriage than women [4,8]. Furthermore, family scholars have argued that marital health advantages are often attenuated among individuals from lower socioeconomic status (SES) backgrounds due to poorer relationship quality [11]. Lower SES groups often face additional stressors and limited access to resources, which can negatively impact the quality of their marital relationships and subsequently diminish the health benefits that typically accompany marriage [12].

Family scholars have developed theoretical models to understand the mechanisms through which marriage can influence health outcomes, [13,14] which may also provide insights into predicting differences in vaccination uptake rates based on marital status. Among the most frequently utilized theories to explain the health advantages associated with marriage is the marital resource model. This model posits that marriage provides unique social, psychological, and economic resources that are beneficial to health [13,15]. These resources, such as social support and access to healthcare, may also play a role in promoting vaccination uptake [16,17]. Moreover, married individuals tend to have larger social networks, [13,15] and these networks can provide valuable health promotion information, including information about vaccines, which may positively impact vaccination rates. Additionally, the economic advantages associated with marriage, such as improved financial resources through pooled income and increased access to healthcare insurance through spousal employment, may contribute to higher vaccination rates among married individuals [13].

Empirical investigation of marital status difference in vaccination has been mostly focused on the influenza vaccine and has provided mixed empirical evidence, [18] with some studies suggesting a higher vaccination rate among married adults when compared to their unmarried counterparts [19,20,21] while other studies finding no such differences [22,23]. Emerging research on COVID-19 vaccination hesitation/acceptance has also revealed mixed evidence on marital status differences [24,25]. Some studies have suggested that married people have higher levels of acceptance of COVID-19 vaccination than unmarried people, [24] while other studies found no significant association between marital status and COVID-19 vaccine hesitancy [25]. Yet, there is little research on whether the COVID-19 vaccination varies across marital status groups in the U.S. or whether these patterns vary by gender or socioeconomic status.

Using data from the National Health and Aging Trends Study (NHATS) 2021, [26] we provide one of the first population-based studies on marital status differences in COVID-19 vaccination among older Americans during the early stage of the pandemic (i.e., the first year after the vaccine first became available). In doing so, we address three major research questions: (1) Does early COVID-19 vaccine uptake vary by marital status among older Americans? (2) Do these patterns vary by gender? and (3) Do these patterns vary by socioeconomic status (proxied by education)? The importance of this study is highlighted by the rapidly growing number of unmarried older adults in the U.S., particularly as the divorce rate among adults aged 50 and older has doubled between 1990 and 2010 [27]. Currently, two out of five Americans aged 65–74 and one out of four Americans aged 75 and older have been divorced [28]. Findings from this study will help health policymakers and practitioners target the most vulnerable subpopulations in order to design effective intervention strategies and public programs to promote vaccination among older adults in order to prepare for future pandemics. 

## 2. Methods

### 2.1. Data and Sample

Data were drawn from the NHATS 2021, which was conducted by the Johns Hopkins University Bloomberg School of Public Health in collaboration with the University of Michigan. NHATS gathers information, through annual in-person interviews, from a nationally representative sample of Medicare beneficiaries aged 65 years and older who live in communities, residential care, or nursing homes within the contiguous U.S. (i.e., excluding Alaska, Hawaii, and Puerto Rico) to foster research that will reduce disability, maximize health and independent functioning, and enhance the quality of life at older ages [29]. NHATS utilizes Medicare’s enrollment database as the sampling frame and oversamples older persons and Black individuals [29]. In 2011, 8245 respondents aged 65 years and older completed the initial (Round 1) interview (71% response rate). Respondents have been reinterviewed annually to document changes over time, with the most recently released follow-up being the 2021 wave (Round 11). A replenishment sample was added in 2015 to maintain its ability to represent the older Medicare population.

**Figure 1:**
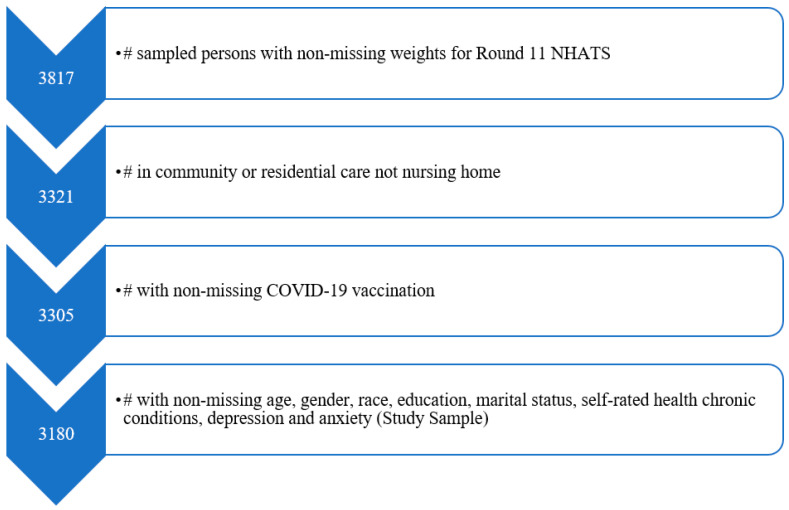
National Health and Aging Trends Study Round 11 and Study Sample Inclusion Criteria.

In this study, we used the NHATS data (*n* = 3817) from Round 11 which was collected from June to November of 2021 to provide measures for COVID-19 vaccination. We excluded nursing home residents from the analysis because they were not eligible for the NHATS sample person (SP) interview where most of our analytic variables were derived. We restricted our analysis to 3180 participants (1846 women and 1334 men) who had complete data for COVID-19 vaccination and covariates (see Figure 1 for exclusion criteria and sample size).

### 2.2. Measures

*Dependent variable. COVID-19 vaccination status* was measured based on the question asking the respondents whether they have been vaccinated for COVID-19 (0 = no, 1 = yes).

*Independent variable. Marital status* included four categories: married (reference, including cohabiting), divorced/separated, widowed, and never married. We combined the married and cohabiting into one group due to the small sample size of cohabitors in our sample (*n* = 63). Previous studies suggested that marriage and cohabitation tended to be similar among older adults [30]. Results from our supplementary analyses (not shown but available upon request) also suggested no statistically significant differences between these two groups in vaccination status. 

*Potential moderators.* We tested *gender* (0 = men, 1 = women) and *education* (0 = lower education with no college, 1 = higher education with some college or college graduate and above) as potential moderators for the association between marital status and vaccination. Specifically, the lower-educated group included high school graduates and those who had no high school diploma. The higher-educated group included those with some college education or a Bachelor’s degree or a graduate or professional degree.

*Control covariates.* We controlled for several covariates that may relate to both marital status and vaccination, including *age* (65–69 [reference], 70–74, 75–79, 80–84, 85–89, 90+), *race-ethnicity* (non-Hispanic White [reference], non-Hispanic Black, Hispanic, and other), *region* (South [reference], Northeast, Midwest, and West), the *importance of religious services* (not so important [reference], somewhat important, and very important) and whether participants reported *walking as the primary mode of transportation* (0 = no; 1 = yes). Moreover, we included additional control measures related to mental and physical health. Physical health measures were comprised of *self-rated physical health* (from 1 = poor/fair to 5 = excellent) and the *number of chronic conditions* out of a total of 10 conditions (heart attack, heart disease, high blood pressure, arthritis, osteoporosis, diabetes, lung disease, stroke, dementia, and cancer). Mental health measures included depressive symptoms and anxiety. *Depressive symptoms* were measured using the 2-item Patient Health Questionnaire (PHQ-2), a validated screening tool for depression [31]. The score ranged from 2 to 8 with higher scores indicating more depressive symptoms and a score greater than or equal to 3 was used as a cutoff to screen for depression, which had been found to have higher sensitivity and specificity than other cutoffs [32]. *Anxiety* was measured using the 2-item Generalized Anxiety Disorder (GAD) and a cutoff of 3 for screening was found to perform well [33]. In addition, we included a measure of whether health issues prevented participants from *attending religious services* (0 = no; 1 = yes). 

### 2.3. Statistical Analyses

We first presented the unweighted frequencies, weighted proportions, and confidence intervals (CIs) for discrete variables, weighted mean, standard error of the mean, and confidence intervals for continuous variables. We then reported *p*-values based on design-corrected F-tests, comparing marital status and other demographic and health measures between the vaccinated and the unvaccinated. For research question 1 (Does early COVID-19 vaccine uptake vary by marital status among older Americans?), we estimated binary logistic regression models and reported unadjusted and adjusted odds ratios (ORs) and 95% CIs for marital status on vaccination, controlling for age, gender, race-ethnicity, education, region, the importance of religious services, attending religious services, self-reported physical health, chronic conditions, depression, and anxiety. For research questions 2 and 3 (Do these patterns vary by gender or socioeconomic status?), we tested the interaction effects of gender by marital status and education by marital status respectively in predicting vaccination. Instead of reporting ORs of the interactions, we followed the guidance of best practices for interpreting nonlinear interactions and reported the effects on the risk and risk difference scales [34]. All estimates used the final analytic weights supplied by NHATS, considering differential responses in the COVID-19 supplement such that standard errors reflected the complex design of NHATS. All analyses were performed using Stata v17. 

## 3. Results

### 3.1. Descriptive Statistics

Table 1 shows weighted descriptive statistics of all analytic variables in the study sample as well as the corresponding 2021 U.S. Census American Community Survey 1-Year Estimates for the represented population [35]. Compared to census estimates, NHATS 2021 respondents were a little older and had a slightly higher proportion of being widowed, but the gender and racial-ethnic distributions were similar to the general population.

Table 2 compares demographic and health characteristics by vaccination status in the study sample. Compared to those who were vaccinated, those who were not vaccinated were more likely to be divorced/separated (22.8% vs. 13.2%) or widowed (32.3% vs. 29.5%), have no college education (57.6% vs. 42.3%), have a higher proportion of reporting depressive symptoms (17.4% vs. 9.6%), and have a lower proportion of reporting very good or excellent health (34.7% vs. 42.8%).

### 3.2. Marital Status Differences in COVID-19 Vaccination 

Table 3 shows unadjusted and adjusted ORs of receiving COVID-19 vaccination by marital status from logistic regression models. Compared to married/cohabiting respondents, divorced/separated respondents had significantly lower odds of receiving COVID-19 vaccination (unadjusted OR = 0.45, 95% CI (0.28, 0.72), *p* < 0.01; adjusted OR = 0.45, 95% CI (0.27, 0.77), *p* < 0.01). The odds of receiving COVID-19 vaccination were not significantly different for the widowed and never married in comparison to the married.

### 3.3. Gender and Educational Differences

**Figure 2:**
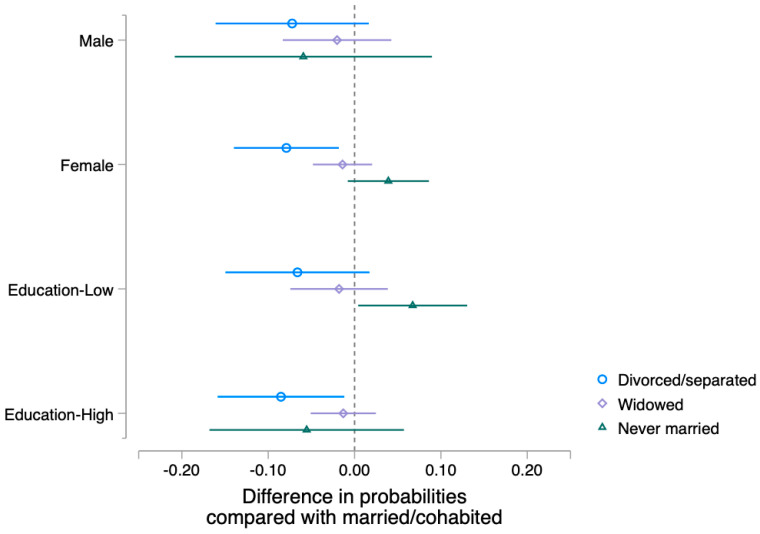
Difference in Probabilities of COVID-19 Vaccination Relative to Married or Cohabited Participants by Gender and Education.

Figure 2 illustrates the differences in the predicted probability of receiving COVID-19 vaccination by marital status by gender and education. Among female respondents, the divorced/separated had an eight-percentage-point-lower probability of receiving COVID-19 vaccination than married/cohabiting respondents, but this difference was not present among male respondents. Similarly, among higher-educated respondents, the divorced/separated had an eight-percentage-point-lower probability of receiving COVID-19 vaccination than married/cohabiting respondents, but this difference was not present among lower-educated respondents. Moreover, among the lower-educated group, never married respondents had a seven-percentage-point-higher probability of receiving vaccination than married/cohabiting respondents, albeit this pattern was not shown among the higher-educated group.

## 4. Discussion

The COVID-19 pandemic has imposed unprecedented burdens on morbidity, mortality, and mental health worldwide. The widespread administration of vaccines and achievement of substantial population coverage are touted by leading clinicians and scientists as a front-line approach in preventative medicine for COVID-19 infections [36]. Yet, COVID-19 vaccine uptake is not uniformly distributed across groups [25,37,38]. The present study is one of the first to examine marital status differentials in receiving vaccine protection against the SARS-CoV-2 virus among elderly U.S. community dwellers. Our analysis of a nationally representative sample of older adults aged 65 and above highlights marital status as an important social factor to predict COVID-19 vaccine uptake.

First, we found that divorced/separated older adults had significantly lower COVID-19 vaccine uptake than their married counterparts during the early stage of the pandemic when the vaccine initially became widely available. Given the high effectiveness of COVID-19 vaccines in protecting people from getting seriously ill, being hospitalized, and dying, [39] divorced/separated older adults who were the least likely to receive the vaccine may be the most vulnerable group during the pandemic. This finding is consistent with our expectation as well as the general literature suggesting major health disadvantages of divorced/separated people relative to their married counterparts [4,40]. This is one of the first studies to extend the health disadvantage of divorce or separation to COVID-19 vaccination and prevention. One possibility is that divorced/separated older adults have more limited health information and knowledge and thus have worse vaccine awareness or greater vaccine hesitancy than their married counterparts [25]. It is also conceivable that divorced/separated people have less access to COVID-19 vaccines than married people, especially during the early stage of the pandemic when the vaccine became available, because they were more likely to live in socioeconomically disadvantaged neighborhoods with limited healthcare facilities [41]. Future research should identify the underlying causes that lead to lower vaccination rates among divorced/separated older adults in order to boost vaccine uptake.

The vaccine disparity between divorced/separated and married older adults further varies by gender and education. Our results suggest that the lower COVID-19 vaccine uptake among divorced/separated older adults relative to married older adults only presented among the higher-educated group but not the lower-educated group. This finding is indeed consistent with previous studies suggesting that marital advantage in health tends to diminish among lower socioeconomic status groups [11]. Moreover, our study demonstrates that the lower COVID-19 vaccine uptake among divorced/separated older adults relative to married older adults only presented among women but not among men. The reason for this gender variation is unclear. Given that previous studies suggest that marital status differences in health are greater for men than for women, [4,8] future studies should analyze other datasets to confirm this pattern and further investigate specific mechanisms that contribute to the gendered patterns in the associations between marital status and COVID-19 vaccine uptake.

Finally, the widowed and never married were not significantly different from the married in COVID-19 vaccination, with only one exception: lower-educated, never married respondents were more likely to receive COVID-19 vaccination than their lower-educated, married counterparts. Although this finding is not expected, it is consistent with a growing number of studies showing no health disadvantage or even advantage among the never-married in comparison to their married counterparts [4,42]. Indeed, a recent study suggested that never-married people were more involved with their communities than other marital status groups, [42] which may help them raise vaccine awareness. Nevertheless, our sample of never-married older adults is small. Future studies should analyze other datasets to confirm the robustness of this finding.

This study has several limitations. First, the COVID-19 vaccine status is based on a self-reported measure in this study. The issue of misreporting cannot be ignored. Second, data derived from NHATS 2021 were collected during the early stage of the pandemic when the COVID-19 vaccine first became available. Our findings are limited to this particular time period of the pandemic. Future studies should investigate how vaccination status changes across marital status groups as the COVID-19 pandemic goes through different stages as more data become available. Finally, although it is beyond the scope of the current study to explore the specific mechanisms that lead to the identified marital status differences in COVID-19 vaccination, it is important for future studies to continue with this endeavor.

## 5. Conclusions

The present study contributes to the existing marriage and health literature by examining COVID-19 vaccination, a crucial public health concern in the context of the pandemic [43,44,45]. Drawing on a nationally representative sample of older adults in the United States, our findings suggest that divorce/separation is a significant social factor associated with COVID-19 vaccination, particularly among women and those with higher levels of education. These results have important health equity implications, suggesting potential disparities in vaccine access and uptake among different marital status groups, which could worsen existing health disparities. Our study highlights the vulnerability of divorced/separated older adults as a population segment with the lowest COVID-19 vaccine uptake. Therefore, future efforts to increase vaccinations should target this group with specific interventions to improve their access and uptake. This could involve education campaigns to increase awareness of the importance of vaccination and efforts to remove barriers to vaccine access. Overall, our study emphasizes the need for targeted interventions to improve vaccine equity and uptake among older adults, with particular attention to the needs of divorced/separated individuals. By addressing the unique needs of this group, we can promote a more equitable distribution of vaccines and contribute to the fight against future pandemics.

## Figures and Tables

**Table 1:** Weighted Descriptive Statistics of Analytic Variables in National Health and Aging Trends Study, 2021.

	Unweighted N (Weighted %)	95% Confidence Interval		U.S. Elderly Population Reference (65 and Older) ^a^
Vaccinated	2867 (90.4%)	(88.5%	92.0%)	94.2% ^e^
Marital Status				
Married	1374 (50.4%)	(48.2%	52.6%)	31,490,252 (56.31%)
Cohabited	63 (2.4%)	(1.8%	3.3%)	N.A. ^b^
Divorced/separated	451 (14.1%)	(12.9%	15.5%)	9,072,576 (16.22%)
Widowed	1177 (29.8%)	(27.9%	31.7%)	11,743,430 (21.00%)
Never married	115 (3.3%)	(2.6%	4.1%)	3,616,455 (6.47%)
Gender				
Male	1334 (44.3%)	(42.0%	46.7%)	25,191,742 (45.07%)
Female	1846 (55.7%)	(53.3%	58.0%)	30,700,272 (54.93%)
Education				
Less than high school	498 (12.8%)	(11.1%	14.6%)	7,015,917 (12.55%)
High school diploma	1032 (31.0%)	(28.3%	33.8%)	16,801,639 (30.06%)
Some college or Bachelor’s degree	653 (22.3%)	(20.5%	24.2%)	24,432,134 (43.71%)
Graduate or prof. degree	997 (34.0%)	(30.7%	37.4%)	7,642,324 (13.67%)
Age				
65 to 69	N/A	N/A	N/A	18,351,785 (32.83%)
70 to 74	430 (28.9%)	(26.8%	31.1%)	15,426,419 (27.60%)
75 to 79	950 (32.8%)	30.6%	35.1%)	9,872,768 (17.66%)
80 to 84	796 (20.1%)	(18.5%	21.8%)	6,278,369 (11.23%)
85 to 89	576 (11.5%)	(10.5%	12.5%)	5,962,673 (10.67%)
90+	428 (6.7%)	(6.0%	7.4%)	N.A. ^c^
Race-ethnicity				
Non-Hispanic White	2296 (80.7%)	(77.9%	83.2%)	41,494,577 (74.24%)
Non-Hispanic Black	648 (8.1%)	(7.1%	9.3%)	5,138,453 (9.19%)
Hispanic	162 (7.6%)	(5.8%	9.9%)	5,045,939 (9.03%) ^d^
Other or multiple	74 (3.6%)	(2.6%	4.9%)	4,213,045 (7.54%)
Region				
South	1274 (40.1%)	(36.8%	41.6%)	
Northeast	486 (15.3%)	(15.4%	19.6%)	
Midwest	831 (26.1%)	(20.4%	24.4%)	
West	589 (18.5%)	(19.7%	22.6%)	
Importance of religious services				
Not so important	916 (28.8%)	(30.5%	35.4%)	
Somewhat important	626 (19.7%)	(18.6%	22.5%)	
Very important	1638 (51.5%)	(43.5%	49.7%)	
Walking as mode of transportation				
No	1630 (51.3%)	(43.6%	49.0%)	
Yes	1550 (48.7%)	(51.0%	56.4%)	
Depression (PHQ2 ≥ 3)	387 (10.3%)	(9.1%	11.8%)	
Anxiety (GAD2 ≥ 3)	310 (9.0%)	(7.7%	10.5%)	
Self-rated physical health				
Poor	142 (3.9%)	(3.1%	4.8%)	
Fair	603 (16.5%)	(15.0%	18.1%)	
Good	1215 (37.6%)	(35.6%	39.6%)	
Very good	944 (31.6%)	(29.5%	33.8%)	
Excellent	276 (10.5%)	(9.0%	12.1%)	
Health prevented attendance of religious services				
No	2822 (88.7%)	(89.9%	92.5%)	
Yes	358 (11.3%)	(7.5%	10.1%)	
Number of chronic conditions	Weighted Mean (SE)			
	2.76 (0.02)	(2.72	2.81)	

^a^ U.S. Census data obtained from data.census.gov (2021: ACS 1-Year Estimates). ^b^ Cohabited and married are combined into one category in subsequent analyses. ^c^ U.S. Census data top-coded at ‘85 and older’. ^d^ Hispanic origin is distinct from race in the 2020 U.S. Census. ^e^ Source: US Census Household Pulse Survey (as of 10 November 2021).

**Table 2:** Comparison between Vaccinated and Unvaccinated in National Health and Aging Trends, 2021.

	Not Vaccinated	Vaccinated	*p*-Value ^a^
	Weighted %	Weighted %	
Marital status			0.003
Married/cohabited	41.9%	54.0%	
Divorced/separated	22.8%	13.2%	
Widowed	32.3%	29.5%	
Never married	2.9%	3.3%	
Gender			0.325
Male	40.8%	44.7%	
Female	59.2%	55.3%	
Education ^b^			<0.001
Less than high school	19.0%	12.1%	
High school diploma	38.6%	30.2%	
Some college or Bachelor’s degree	23.1%	22.2%	
Graduate or prof. degree	19.3%	35.5%	
Age			0.434
70 to 74	25.1%	29.3%	
75 to 79	36.2%	32.4%	
80 to 84	19.5%	20.2%	
85 to 89	11.0%	11.5%	
90+	8.2%	6.5%	
Race			0.390
White	77.1%	81.1%	
Black	8.5%	8.1%	
Hispanic	9.2%	7.4%	
Other or multiple	5.3%	3.4%	
Region			
South	44.6%	38.6%	0.408
Northeast	13.3%	17.9%	
Midwest	20.5%	22.5%	
West	21.5%	21.0%	
Importance of religious services			
Not so important	26.9%	33.6%	0.004
Somewhat important	15.7%	21.0%	
Very important	57.4%	45.4%	
Walking as a mode of transportation			
No	54.2%	45.4%	0.029
Yes	45.8%	54.6%	
Depression (PHQ2 ≥ 3)	17.4%	9.6%	<0.001
Anxiety (GAD2 ≥ 3)	10.9%	8.8%	0.320
Self-rated physical health			0.039
Poor	4.1%	3.9%	
Fair	17.6%	16.4%	
Good	43.6%	36.9%	
Very Good	22.0%	32.6%	
Excellent	12.7%	10.2%	
Health prevented attendance of religious services			
No	86.8%	91.7%	0.011
Yes	13.2%	8.3%	
Number of chronic conditions	Mean (SE)	Mean (SE)	
	2.78 (0.12)	2.76 (0.02)	0.907

^a^ Based on design-corrected F test. ^b^ Subsequent analyses combines ‘Bachelor’s degree or less’ and ‘Graduate or prof. degree’ into a single category.

**Table 3:** Unadjusted and adjusted odds ratios (ORs) for marital status on COVID-19 vaccination.

Marital Status	Unadjusted OR (95% CI) ^a^	Adjusted OR (95% CI) ^b^
Married/cohabiting Ref.		
Divorced/separated	0.45 (0.28–0.72)	0.45 (0.27–0.77)
Widowed	0.71 (0.49–1.02)	0.80 (0.53–1.21)
Never married	0.88 (0.33–2.35)	0.93 (0.33–2.60)

^a^ Estimated using NHATS analytic sampling weights and sample design strata. ^b^ Estimated using NHATS analytic sampling weights and sample design strata adjusting for age, gender, race, education (some college or college graduate vs. lower education levels), region, the importance of religious services, attending religious services, walking as the primary mode of transportation, self-rated physical health (poor and fair are collapsed to one group), number of chronic conditions, depression, and anxiety.

## Data Availability

The data is publicly available at https://www.nhats.org (accessed on 7 June 2023).
